# Recurrent Inflammatory Intratarsal Keratinous Cyst Following an Incomplete Excision: A Case Report

**DOI:** 10.7759/cureus.114772

**Published:** 2026-08-19

**Authors:** Ryo Kamide, Tatsuya Yunoki, Kenicih Hirabayashi, Atsushi Hayashi

**Affiliations:** 1 Department of Ophthalmology, University of Toyama, Toyama, JPN; 2 Departments of Ophthalmology, University of Toyama, Toyama, JPN; 3 Department of Diagnostic Pathology, University of Toyama, Toyama, JPN

**Keywords:** chalazion, incomplete excision, intratarsal keratinous cyst, recurrence, secondary inflammation

## Abstract

Intratarsal keratinous cyst (IKC) is a rare benign lesion arising within the tarsal plate that is frequently misdiagnosed as a chalazion. An IKC's incomplete excision often leads to multiple recurrences, but cases accompanied by acute inflammation or pain remain uncommon. We report a rare case of an IKC presenting with recurrent painful inflammatory episodes following incomplete surgical removal. An 80-year-old male presented with a firm, painless mass in his left upper eyelid. Eyelid eversion revealed a whitish lesion through the palpebral conjunctiva. Under the clinical diagnosis of a chalazion, an initial incision and curettage were performed; however, the cyst wall was not completely extirpated. The histopathological examination of the specimen confirmed IKC, showing no inflammatory cell infiltration or granulation tissue. Postoperatively, the patient experienced three painful inflammatory recurrences at the same site over a two-year period, each temporarily resolving with antimicrobial therapy. To achieve a definitive cure, complete surgical excision including the entire cyst wall was performed after the third recurrence. The histopathological analysis of the completely excised recurrent lesion revealed prominent inflammatory cell infiltration and granulation tissue formation surrounding the residual cyst wall. No recurrence has been observed for more than 12 months postoperatively. An incomplete excision of an IKC can trigger secondary inflammation and subsequent intractable, painful recurrences. Clinicians should consider IKC in the differential diagnosis of recurrent chalazion-like lesions, and a prompt histopathological evaluation with complete extirpation of the cyst wall is essential for a definitive cure.

## Introduction

An intratarsal keratinous cyst (IKC) is a clinical entity first proposed by Jakobiec et al. in 2010, representing a reclassification of a subset of lesions previously diagnosed as recurrent chalazion or subcutaneous cyst [1]. An IKC is a keratinous cyst arising within the tarsal plate; its wall consists of stratified squamous epithelium, and the cavity is filled with laminated keratin and milky atheromatous material resembling a thick, "tofu-like" substance, which provides a critical intraoperative diagnostic clue [1,2]. Histopathologically, an IKC is characterized by a cyst wall lined with stratified squamous epithelium with a granular layer, alongside keratotic contents, demonstrating a histological appearance similar to that of an epidermoid cyst [1,2]. Clinically, IKCs occur predominantly in the upper eyelid, presenting as a firm, painless mass fixed to the tarsal plate. Mobility with respect to the overlying skin is preserved, and upon eyelid eversion, the lesion is frequently observed from the conjunctival side as a whitish or bluish-white cyst [2,3].

IKCs are frequently misdiagnosed as chalazia and treated with incision and curettage without histopathological examination, leading to an underestimation of their true incidence. Following incomplete excision, IKCs have a characteristic tendency to recur at the same anatomical site, typically manifesting as a slow, painless, non-inflammatory keratin re-accumulation. Reports of IKC cases accompanied by acute inflammation or pain remain extremely rare [1,2,4]. To address this clinical knowledge gap, we report a highly unusual case documenting the clinicopathological evolution of an IKC that presented with recurrent inflammatory episodes following an initial incomplete excision, along with a brief review of the relevant literature.

## Case presentation

**Figure 1:**
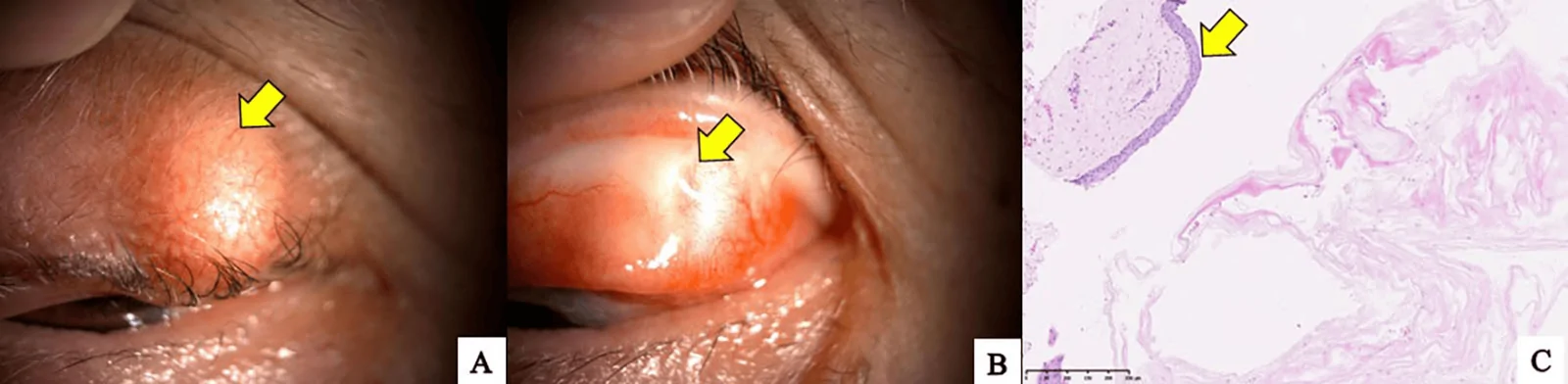
Initial clinical and histopathological findings of the patient, an 80-year-old male. (A) Slit-lamp photograph showing a firm mass within the patient's left upper tarsal plate (yellow arrow). (B) Slit-lamp photograph with eyelid eversion, showing a whitish lesion visible through the palpebral conjunctiva (yellow arrow). (C) Histopathology of the initially excised specimen (hematoxylin and eosin, original magnification ×12) showing a keratin-filled cyst lined by stratified squamous epithelium (yellow arrow) without inflammatory cell infiltration or granulation tissue. Scale bar = 250 μm.

An 80-year-old male was referred to our department with the chief complaint of a mass in his left upper eyelid. The initial examination identified a firm, painless mass measuring approximately 10 mm in size within the tarsal plate, localized toward the lateral aspect of the left upper eyelid. There was no erythema, swelling, or thinning of the overlying skin, no visible punctum, and occlusion of the meibomian gland orifices was absent. The lesion was located away from the eyelid margin, and a whitish lesion was visible through the palpebral conjunctiva (Figures 1A, 1B). 

Under the clinical diagnosis of a chalazion, initial incision and curettage were performed as planned. Intraoperatively, thick, milky atheromatous material resembling a "tofu-like" substance [2] was observed, raising suspicion of an alternative diagnosis; however, the procedure was completed with standard curettage, leaving the potential cyst wall unexcised. Hematoxylin and eosin staining of the excised specimen revealed a cyst structure lined with stratified squamous epithelium with a granular layer and containing laminated keratin in the lumen; based on the strict intratarsal location, classic "tofu-like" contents [2], and typical histology [1,2], the absence of inflammatory cell infiltration and granulation tissue led to the definitive diagnosis of an IKC (Figure 1C).

**Figure 2:**
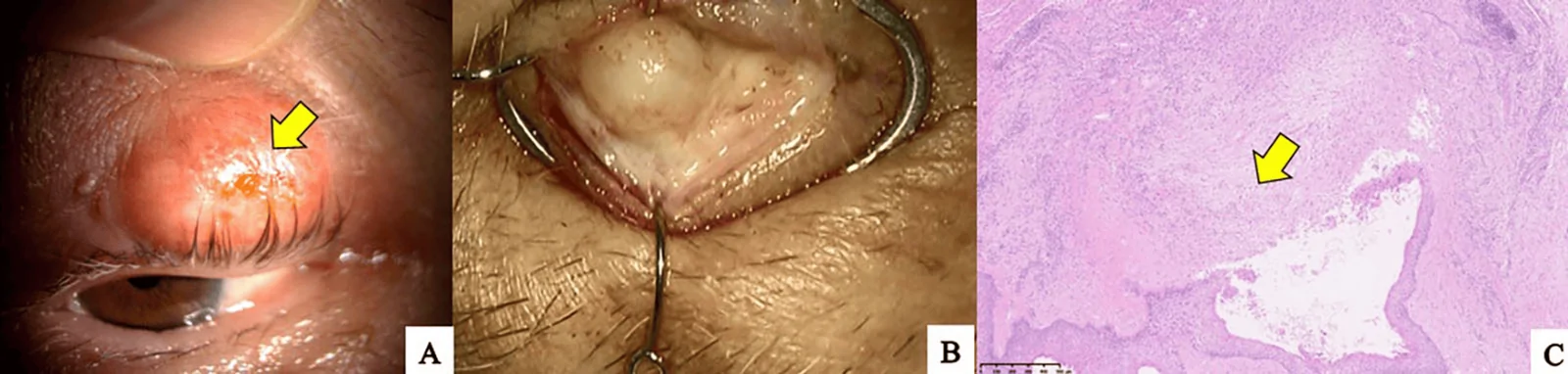
Clinical, intraoperative, and histopathological findings of the recurrent lesion. (A) Slit-lamp photograph at the third recurrence, showing prominent erythema and swelling of the left upper eyelid (yellow arrow). (B) Intraoperative photograph of the complete excision, including the entire cyst wall. (C) Histopathology of the excised recurrent specimen (hematoxylin and eosin, original magnification ×4.6), showing prominent inflammatory cell infiltration and granulation tissue (yellow arrow) around the residual cyst wall. Scale bar = 500 μm.

However, postoperatively, the patient experienced recurrent painful inflammatory episodes at the same site, with a total of three recurrences observed over a two-year period, representing a highly unusual clinical presentation, in contrast to the typical slow, painless, non-inflammatory enlargement following incomplete excision. Although each recurrence (including the third episode, shown in Figure 2A) was temporarily resolved with antimicrobial therapy, given the frequent inflammatory recurrences and the definitive histopathological diagnosis of IKC, a complete surgical excision including the entire cyst wall was ultimately performed after the third recurrence for a definitive cure (Figure 2B). The histopathological examination of the completely excised specimen revealed inflammatory cell infiltration and the formation of granulation tissue around the cyst wall (Figure 2C). The patient has been followed up for more than 12 months postoperatively, with no recurrence observed to date. 

## Discussion

Based on our literature search, no previous cases of IKC have been reported to exhibit recurrent inflammatory episodes after an initial histopathological diagnosis. IKCs are known to be frequently misdiagnosed as a chalazion, leading to cases in which only incision and drainage or curettage of the contents is performed without a histopathological examination, resulting in multiple recurrences [1,2,4]. In the present case, the patient had remained asymptomatic until the initial presentation, and the initial specimen exhibited no baseline inflammatory changes. However, subsequent recurrent inflammation developed following the initial procedure. This clinical course suggests that the initial surgical trauma and incomplete curettage likely disrupted the remaining cyst wall, exposing the residual keratin contents to adjacent tarsal tissues. This mechanical tissue injury and secondary foreign-body response to the residual keratin-rather than the intrinsic nature of the IKC itself-served as the primary trigger for the subsequent granulomatous inflammation. While the histopathological findings support a secondary inflammatory process, mechanical factors such as tissue distortion or local nerve irritation during cyst expansion may also have contributed to the recurrent pain. Furthermore, surgical manipulation during the initial curettage might have caused localized damage to adjacent tarsal structures, including the meibomian glands, which could have fueled the secondary inflammatory response; nevertheless, the marked granulomatous reaction surrounding the residual cyst wall in our resected specimen strongly indicates that the exposed keratinous material played the central role. Although the histopathological presence of a granular layer and laminated keratin in our initial specimen overlapped with features of a conventional epidermoid cyst [1,2], the strict intratarsal location and characteristic "tofu-like" contents [2] provided sufficient clinicopathological evidence to confirm the diagnosis of IKC without requiring additional immunohistochemical studies. Therefore, complete surgical excision, rather than simple curettage, is essential when treating lesions with atypical clinical findings to avoid intraoperative cyst disruption and subsequent secondary inflammation.

The scarcity of such reports may be attributed to the fact that a histopathological examination is frequently omitted during the initial treatment. In clinical practice, contents obtained from incision and drainage for a suspected chalazion are not uncommonly discarded without a pathological evaluation. Consequently, even when subsequent inflammatory recurrences occur, these lesions may be mismanaged as a refractory or infected chalazion. IKC should thus be included in the differential diagnosis when a firm, painless tarsal mass is observed, particularly one located away from the eyelid margin through which a whitish lesion is visible via the palpebral conjunctiva [2,3]. Moreover, when recurrent inflammatory episodes occur at the identical site after incision or curettage, clinicians should consider the possibility of a residual IKC and promptly plan a histopathological evaluation along with complete extirpation, including the cyst wall [2,4].

## Conclusions

In conclusion, although IKC is a rare clinical entity, clinicians should be aware that incomplete excision can trigger intractable, painful inflammatory recurrences. Early suspicion of a possible IKC based on characteristic clinical findings, followed by a definitive histopathological diagnosis and complete surgical removal of the cyst wall, is essential for a successful outcome.
